# An oxygen enrichment device for lowlanders ascending to high altitude

**DOI:** 10.1186/1475-925X-12-100

**Published:** 2013-10-09

**Authors:** Guanghao Shen, Xiaoming Wu, Chi Tang, Yili Yan, Juan Liu, Wei Guo, Da Jing, Tao Lei, Yue Tian, Kangning Xie, Erping Luo, Jianbao Zhang

**Affiliations:** 1 School of Biomedical Engineering, Fourth Military Medical University, Xi’an, Shaanxi 710032, People's Republic of China; 2 Key Laboratory of Biomedical Information Engineering of Ministry of Education, School of Life Science and Technology, Xi’an Jiaotong University, Xi’an, Shaanxi 710049, People's Republic of China

**Keywords:** Medical devices, Hypoxia, Oxygen enrichment membrane, High altitude

## Abstract

**Background:**

When ascending to the high altitude, people living in low altitude areas will suffer from acute mountain sickness. The aim of this study is to test the hypothesis that whether an oxygen concentration membrane can be made and used to construct a new portable oxygen enrichment device for individuals in acute exposure to the high altitude.

**Methods:**

The membrane was fabricated using vinylsiloxane rubber, polyphenylene oxide hydrogen silicone polymers, chloroplatinic acid and isopropyl alcohol. The membrane was assembled in a frame and the performance was tested in terms of concentration of oxygen, flow rate of oxygen enriched air, pressure ratio across the membrane and ambient temperature. Furthermore, the oxygen concentration device was constructed using the membrane, a DC fan, vacuum pump and gas buffer. A nonrandomized preliminary field test was conducted, in which eight healthy male subjects were flown to Tibet (Lhasa, 3,700 m). First, subjects wore the oxygen enrichment device and performed an incremental exercise on cycle ergometer. The test included heart rate (HR), saturation of peripheral oxygen (SpO_2_) and physical work capacity (PWC). Then, after a rest period of 4 hours, the experimental protocol was repeated without oxygen enrichment device.

**Results:**

The testing showed that the membrane could increase the oxygen concentration by up to 30%. Simulation test indicated that although the performance of the oxygen enrichment device decreased with altitudes, the oxygen concentration could still maintain 28% with flow rate of enriched air 110 cm^3^/s at 5000 m. The field test showed that higher SpO_2_, lower HR, and better PWC (measured by the PWC-170) were observed from all the subjects using oxygen enrichment device compared with non-using (*P* < 0.01).

**Conclusions:**

We concluded that the new portable oxygen enrichment device would be effective in improving exercise performance when ascending to the high altitude.

## Introduction

Qinghai-Tibet Plateau is the highest plateau of the world with an average altitude of over 4,500 m, which is called the roof of the world. In recent years, with the opening of Qinghai-Tibet railway in China, more and more people who live in low altitude areas ascend to high altitude for science investigation, tour or business. Many of them suffer from acute mountain sickness, experiencing hypoxia which is caused by the low partial pressure of oxygen at high altitudes [1,2]. The most common symptoms include headache, nausea, poor appetite, fatigue, insomnia and dizziness, which often occur within the first three days of arrival at high altitude [3]. Moreover, a reduction in exercise capacity on ascent to altitude is universal, it happens to all lowlanders [4]. This problem may severely affect the physical and mental state, hence deteriorate work efficacy for the travelers and workers.

To diminish the risk of acute mountain sickness (AMS), numerous instruments have been developed, including portable hyperbaric chamber [5-7], bottled oxygen [8], and oxygen enrichment room [2]. The oxygen provided by these instruments elevates the oxygen concentration and lowers the equivalent altitude [9,10], which can treat mild AMS. However, none of them can be easily used by individuals for a long time. To overcome the limitations, we have developed a long-lasting and portable anti-hypoxia equipment, the oxygen-increased respirator (OIR) for individuals [11]. While showing improved performance, the OIR suffers from its own limitation that it needs additional pressure to compress the air in order to raise the oxygen density without increasing the oxygen concentration (percentage of oxygen in volume). This sometimes causes irritation when air temperature is low [11].

By raising the concentration of oxygen in the air, the oxygen partial pressure can be increased and the hypoxia effects of the high altitude can be reduced [2]. It has been shown that increasing the oxygen concentration of the air by 1% (e.g. from 21% to 22%) results in a reduction of equivalent altitude of about 300 m [12]. So, providing lowlanders with oxygen enriched air (the oxygen concentration >21%) would improve their performance [13]. Because the output enriched air has higher concentration, there is no need to apply compression to the air before being delivering into nose, which should comfort the users.

The oxygen enrichment membrane is a kind of gas separation membrane, which can be used to obtain oxygen enriched air because the oxygen permeates the membrane faster than nitrogen. It was reported that the oxygen concentration of the air can accumulate up to 40% [14]. It has been widely used in industry, such as steel, engine, and chemistry, in order to induce the complete combustion, save energy and reduce pollution [15].

It is therefore natural to hypothesize that such oxygen enrichment membrane could be used to develop a portable oxygen enrichment device that would help travelers ease their discomforts when ascending to highland. Oxygen enrichment membranes were custom-made and tested for various pressure differences and ambient temperatures. In addition, oxygen enrichment devices were invented and investigated in a hypobaric chamber for various simulated altitudes. Furthermore, a self-controlled study in Lhasa city (altitude: 3700 m), Tibet was performed to examine the actual effects for human subjects.

## Materials and methods

### Ethics statement

The study was approved by the Institutional Ethical Review Board at the Fourth Military Medical University, Xi’an, China.

### Oxygen enrichment membrane

Vinylsiloxane rubber and polyphenylene oxide (6:4 in mass) were dissolved into solvent of cyclohexane in room temperature. In addition, hydrogen silicone polymers, chloroplatinic acid and isopropyl alcohol (1:2:2.5 × 10^-5^ in amount of substance) were dissolved in the above solution. After reaching equilibrium, the solution was coated on the surface of the substrate of polysulfone membrane. The coated membrane was heated before it is available as the oxygen enrichment membrane for later use (patent pending, No. 201210309573.3).

**Figure 1:**
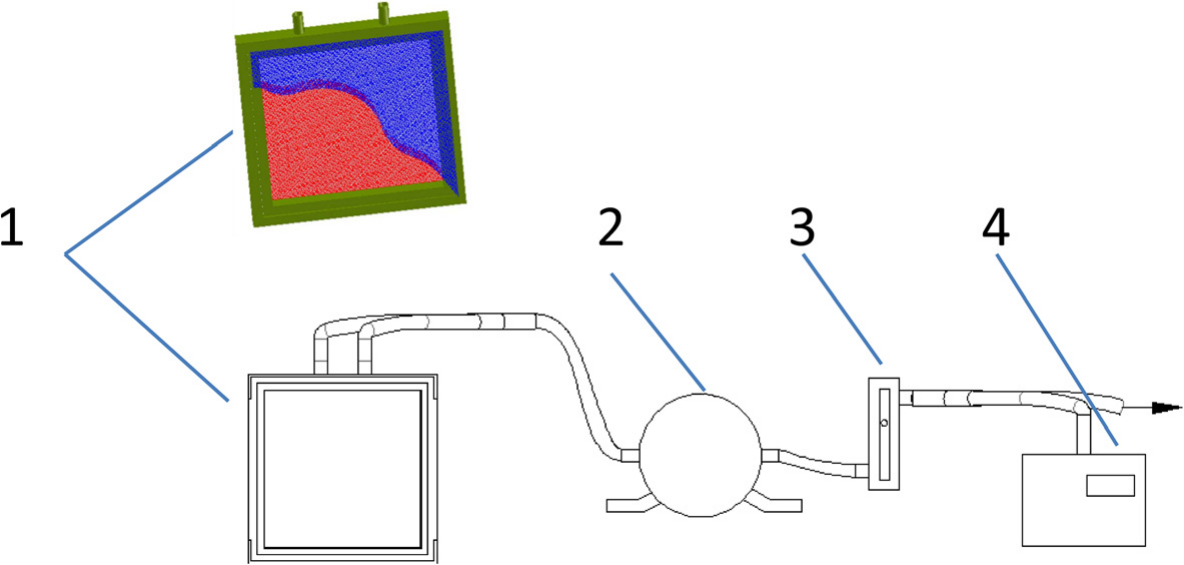
**An oxygen enrichment membrane unit testing system.** The system contains 1) membrane unit; 2) vacuum controller; 3) flow meter; 4) oxygen analyzer. The Green rectangular framework supports the whole structure. The blue part is a piece of membrane (another invisible piece is on the opposite side), which is torn apart to show the supporting nylon layers (red). The red part is the multiple nylon layers which lie in the middle and support the membrane. Two outlets (top) can be used to suck air by a vacuum pump (not shown).

An oxygen enrichment membrane unit (Figure 1) included a rectangular frame that supported two pieces of oxygen enrichment membrane (8.5 cm × 8.5 cm each) between which, there are multiple layers of nylon net. A vacuum controller with digital display (ANB, Xin Wei Cheng, Co., Ltd, Chengdu, China) is used to suck oxygen enriched air out from within the two pieces of membrane. The vacuum degree inside the membrane unit can be clamped at various values by the vacuum controller, and the pressure differences across the membrane can then be calculated by baric pressure minus the vacuum degree. Ambiance temperatures (-10°C–50°C) were simulated in an temperature-controlled chamber. Flow rate of oxygen was measured by a glass rotor flow meter (LZB-6 M, Ruiming Instrument, Changzhou, China). Enriched air was analysed by an oxygen analyzer (Oxymat 61, Siemens, Germany). To test the membrane, concentration of oxygen and flow rate of enriched air were examined at various pressure difference across the oxygen enrichment membrane at altitude of 400 m at temperature 25°C. In addition, concentration of oxygen and flow rate of oxygen enriched air were also measured at various temperatures at altitude of 400 m with pressure difference of 70 kPa.

### Oxygen enrichment device

**Figure 2:**
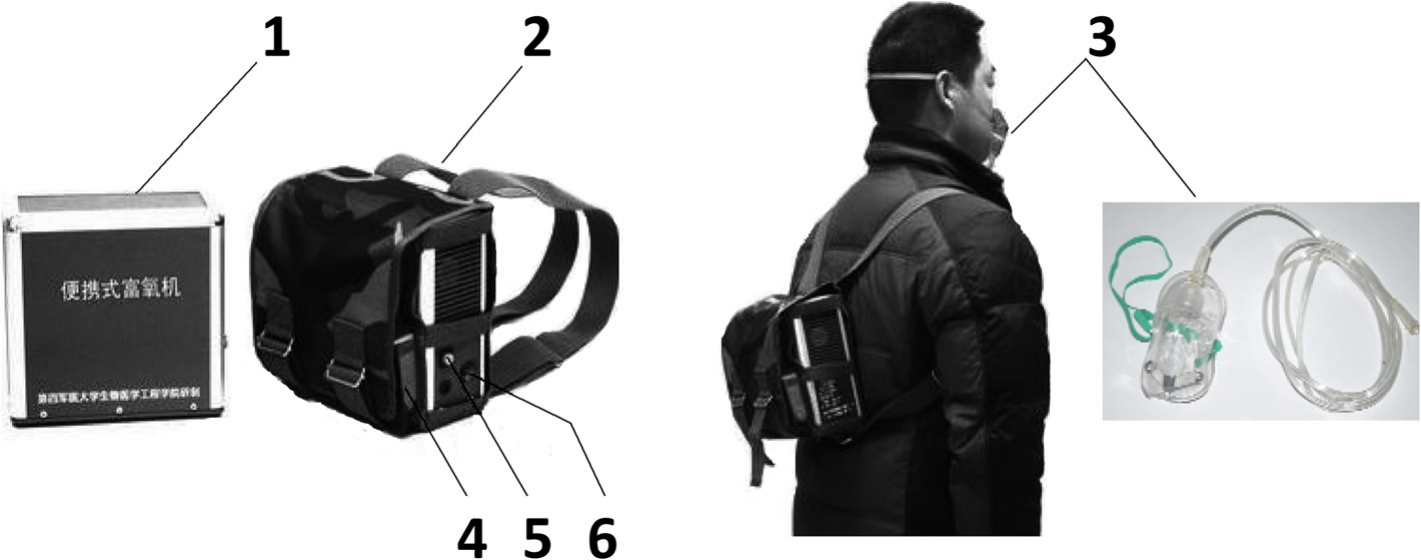
**Production of oxygen enrichment device.** 1) machine body; 2) bag; 3) disposable medical oxygen mask; 4) battery; 5) switch; 6) joint of oxygen enriched air outlet.

**Figure 3:**
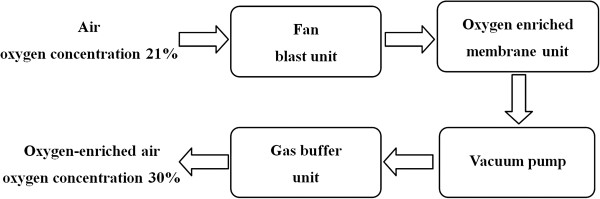
Schematic diagram of the oxygen enrichment device.

Based on the oxygen enrichment membrane, the portable oxygen enrichment device for individuals was invented (Figure 2). The body of the device is composed of four parts: a small DC fan (for ventilation), oxygen enrichment membrane units (16 units in parallel), a vacuum pump (non-oil piston pump), and a buffer (a cylinder, 40 mm in diameter, and 50 mm in height). Since piston pump produces unstable air flow and noises, the buffer is designed to avoid both of these disadvantages. The device can provide oxygen enriched air to respiratory tract by using a disposable medical oxygen mask. The total weight is 2.7 kg, and the dimensions of machine body are 200 mm × 180 mm × 84 mm. For convenience of carrying, it can use rechargeable Li-ion batteries (11.1 V, 7800 mAh, 0.6 kg), which can work continuously for more than 2 hours (Figure 3). With this vacuum pump loading the membrane units, the pressure difference across the membrane was 90 kPa at 1 atm and 25°C. In this study, 8 such devices were fabricated.

### Performance in hypobaric chamber

Eight oxygen enrichment devices were placed inside a hypobaric chamber situated in the school of aerospace medicine at the fourth military medical university. Different altitudes were simulated (400 m, 2000 m, 3000 m, 3500 m, 4000 m, 4500 m, 5000 m, and 5500 m). Concentration of oxygen was measured by an oxygen analyzer. Flow rate of oxygen enriched air at the outlet of the device was measured by the glass rotor flow meter.

### Field test in Lhasa

Eight young men (22–24 years old, Han nationality) in Xi’an city (400 m) were screened based on questionnaire before ascending to Lhasa. Exclusion criteria included cardiac illness, diuretic use, chronic medical conditions, or previous experience of AMS. After signing informed consent forms, they were flown to Lhasa for the first time.

On arrival, all the subjects were asked to have a rest for 2 hours and prepare for the test. First, the subjects were asked to sit down in chairs. heart rate (HR) and saturation of peripheral oxygen (SpO_2_) at rest state were recorded by a multi-parameter patient monitor (IntelliVue MP70, Philips, Eindhoven, Netherlands). After that, the subjects were asked to use the oxygen enrichment devices for 30 minutes. HR and SpO_2_ were recorded again.

Next, to obtain the PWC (physical work capacity)-170 [1,11], subjects performed consecutive workloads on a cycle ergometer (839E, Monark Exercise AB, Sweden) with the oxygen enrichment devices. Subjects were asked to keep the ergometer running at around 60 rpm. Loaded power of the ergometer was initially set to 50 W, then increased by a step of 50 W per 3 minutes until reaching 200 W [16]. A linear regression was conducted and the value of power at HR = 170 beat/min was then predicted by the regression equation. The PWC-170 test was repeated without the devices after 4 hours of resting.

### Statistical analysis

All of the data were shown as means ± SD. SPSS software (ver. 13.0, SPSS Inc., Chicago, IL, USA) was used to perform statistical tests. The paired *t*-test was applied for comparison of HR, SpO_2_ and PWC-170 between two groups after normality test (Shapiro-Wilk test). Exponential fitting was applied to reveal the relationship of flow rate and concentration with respect to pressure ratio. Pearson correlation coefficient was applied to indicate the correlation between parameters (temperature, concentration, flow rate and altitude)*. P* < 0.01 was considered statistically significant.

## Results

### Characteristics of the oxygen enrichment membrane

**Figure 4:**
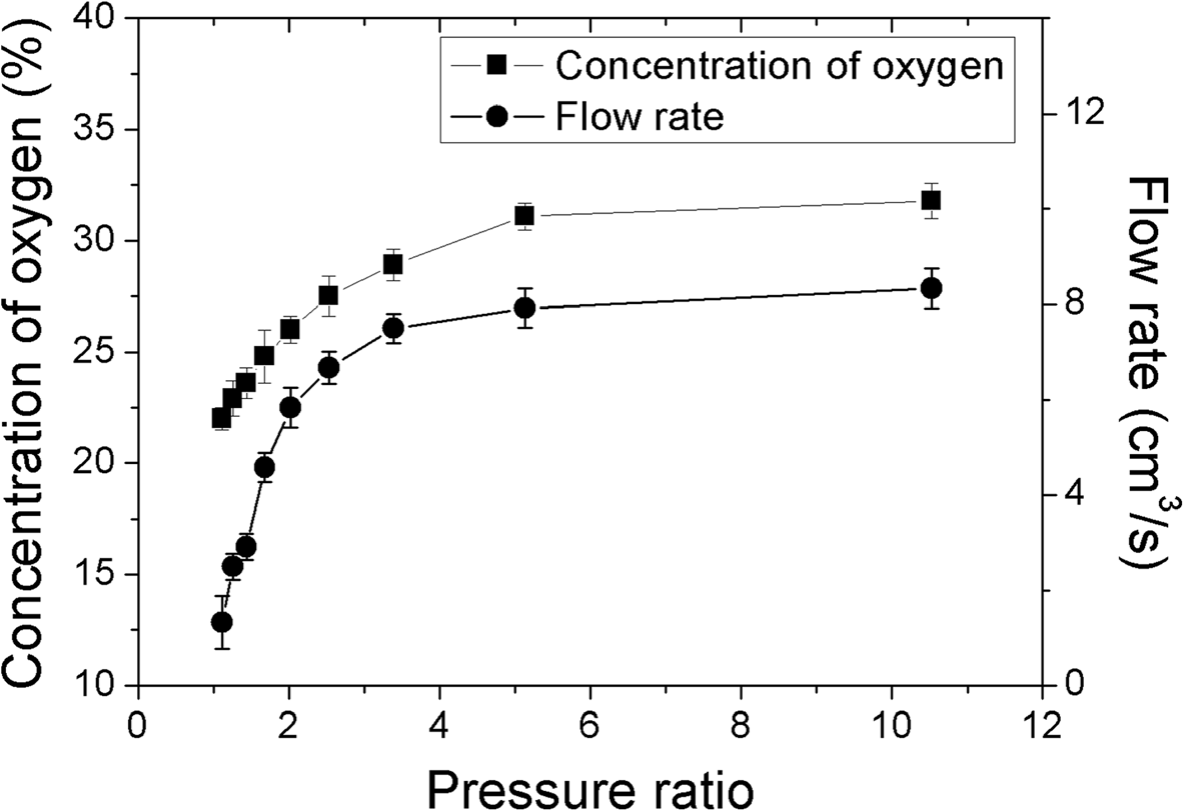
Concentration of oxygen and flow rate of oxygen enriched air as functions of the pressure ratio across the oxygen enrichment membrane at altitude of 400 m at temperature 25°C.

The relationships of concentration of oxygen and flow rate of oxygen enriched air as functions of pressure ratio across the membrane of the unit were shown in Figure 4. The exponential fitting of concentration of oxygen vs. pressure ratio was *y* = ‒ 18.41 * exp(‒ x/1.79) + 31.92 (adjusted R^2^ *=* 0.998, ANOVA, *P* < 0.01). In the mean time, the exponential fitting of flow rate of oxygen enriched air vs. pressure ratio was *y* = - 23.17 * exp(‒ x/0.92) + 8.16 (adjusted R^2^ *=* 0.915, ANOVA, *P* < 0.01).

**Figure 5:**
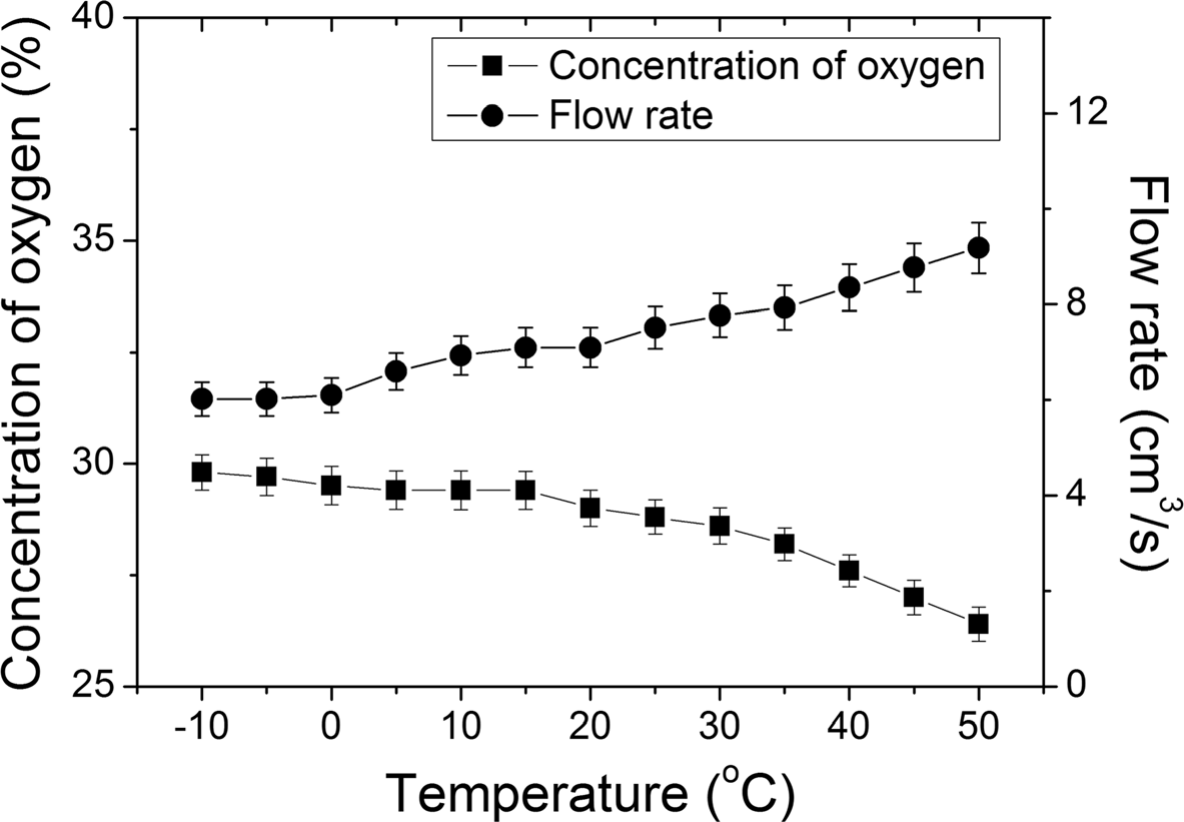
Concentration of oxygen and flow rate of oxygen enriched air as functions of temperature at altitude of 400 m with pressure difference of 70 kPa.

In addition, the concentration of oxygen was reversely correlated with temperature (n = 8; Pearson correlation coefficient, R = -0.932, *P* < 0.01) while the flow rate of oxygen enriched air was positively correlated with temperature (n = 8; Pearson correlation coefficient, R = 0.988, *P* < 0.01); see Figure 5.

### Effects of different altitudes on the oxygen enrichment device

**Figure 6:**
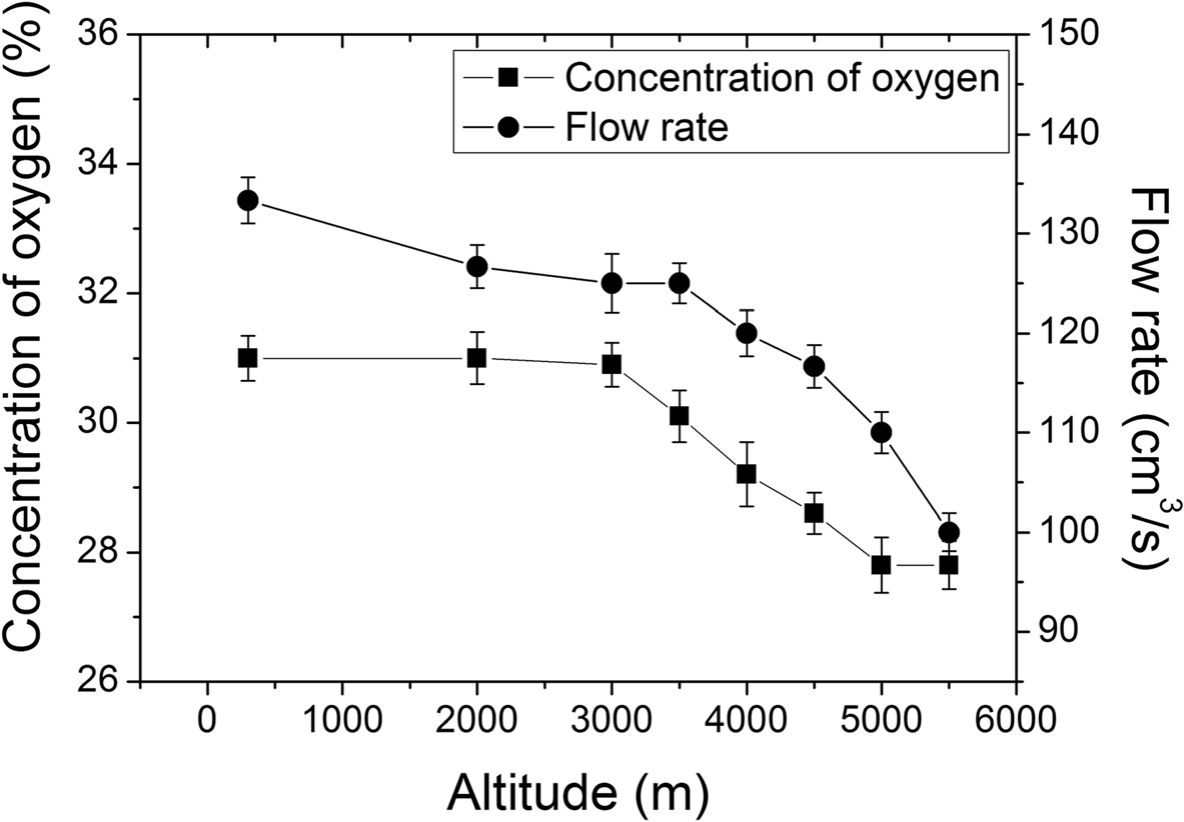
**Simulation in hypobaric chamber.** The concentration of oxygen and flow rate drop as altitude is increasing. The pressure difference between the input and output of the device was set to 90 kPa.

The concentration of oxygen and the flow rate of oxygen enriched air at the outlet of 8 oxygen enrichment devices were found inversely correlated with the simulated altitudes in the hypobaric chamber (R = -0.892, -0.903 respectively, *P* < 0.01; Figure 6).

### Field test of the oxygen enrichment device at Lhasa

The concentration of oxygen of device can be increased to 30%, which is examined by oxygen analyzer at 3,700 m. Its air flow rate of oxygen enriched air can reach more than 7 L/min, which is examined at the same altitude.

The results showed that the subject with oxygen enrichment device had a higher SpO_2_ and lower heart rate at rest than the same subject without devices (*P* < 0.01). Moreover, based on the consecutive workloads test, the power output of the subject with oxygen enrichment device was better than that when without oxygen device (*P* < 0.01, Table 1).

**Table 1:** **Changes in HR, SpO**_**2**_**, and PWC-170 at 3,700 m (Mean ± SD,** n **= 8)**

	**Rest**		**Exercise**
	**HR (beat/min)**	**SpO**_**2**_**(%)**	**PWC-170 (W)**
Without device	83.00 ± 2.07	87.50 ± 1.51	201.26 ± 6.46
With device	75.00 ± 2.73^*^	95.75 ± 1.04^*^	230.43 ± 6.81^*^

Note: *P<0.01. 'Without device’ vs. 'With device’. PWC-170 is the predicted power at HR = 170 beat/min, calculated from linear regression, see Materials and methods for detail.

## Discussion

To reduce the risk of AMS when ascending to high altitude, many medical devices or facilities have been invented, including bottled oxygen, portable hyperbaric chamber and oxygen enrichment room. These devices can raise the oxygen concentration and reduce the equivalent altitude, which can be used to treat mild AMS [12,17]. In this study, we have applied an alternative way by inventing a new oxygen enrichment device by utilizing custom-made oxygen enrichment membrane.

We have shown that, following the increase of the pressure across the membrane, the concentration of oxygen and the flow rate of oxygen enriched air are linearly correlated. Therefore, it is possible to design a device by multiple pieces of parallelized membranes with appropriate pressure via a vacuum pump. By adjusting the pressure and area of the membrane, usable enriched air with appropriate concentration of oxygen and flow rate of the oxygen can be achieved. Such oxygen enrichment device has been manufactured and tested in hypobaric chamber and in Lhasa at high altitude (3700 m). As a portable, durable, and convenient device, the oxygen enrichment device can increase oxygen concentration from 21% to 30% at altitude below 400 m. Its flow rate of oxygen enriched air can reach 116 cm^3^/s.

The field test in Lhasa has shown that the subjects performed with significant improvements in HR, SpO_2_ and PWC-170 wearing the device comparing to non-device conditions. SpO_2_, the indirect measure of the ratio of oxyhemoglobin to the total concentration of hemoglobin present in the blood, serves as an important indicator of oxygen supply for the body, which is measured by a pulse oximetry at fingertip. At high altitude, the low partial pressure of oxygen stemming from the reduced barometric pressure caused less oxygen diffuse into the capillaries within the lungs, finally caused hypoxia and low SpO_2_. A higher level of SpO_2_ should protect cardiopulmonary function during acute exposure to low atmospheric pressure, and may also play an important role in reducing heart overload. Heart rate, having a close relationship to oxygen consumption, increases following ascent to high altitude. The lowered heart rate by wearing the device indicated improved oxygen consumption.

The PWC-170 has also been shown to be a valid tool both in low altitude and high altitude areas [11,18]. The performance improvement of the subjects wearing the device may be explained by the oxygen increase in the air they breathed, which thereafter escalated the partial pressure of oxygen in blood after oxygen diffuses into the capillaries within the lungs.

The current device have overcome the limitation of the previous OIR [11] in that the air pressure delivered into the user’s nasal mask is only slightly higher than local barometric pressure with moderate elevated temperature, which reduces the discomfort of users.

One limitation of the current experimental design in field test is that all the subjects performed tests with oxygen enrichment device first. In future work, more independent experiments concerning the order should be designed. Another limitation is that the disposable medical oxygen masks without reservoir bag are employed as the patient interface, which may be sufficient during exhalation but insufficient during inhalation. To improve the efficiency, a mask with the right volume of reservoir bag should be matched to our device.

## Conclusion

The oxygen enrichment device can not only increase the level of SpO_2_ and physical capacity, but also reduce the heart rate, which can play an important positive role in the protection of people when ascending to high altitude before being acclimatized.

## Abbreviations

HR: Heart rate; SpO2: Saturation of peripheral oxygen; PWC: Physical work capacity; AMS: Acute mountain sickness; OIR: Oxygen-increased respirator.

## Competing interests

The authors declare that they have no competing interests.

## Authors’ contributions

EL, JZ: conceived and designed the study, was involved in funding application, carried out data acquisition, analysis and interpretation, drafted and revised the manuscript. KX: participated in the coordination of the research group, participated in the writing and revision of the manuscript. GS, XW, CT: participated in the experimental tests, drafted and revised the manuscript. YY, JL, WG: participated in the experimental tests, drafted and revised the manuscript. DJ: participated in the experimental tests, carried out data acquisition. TL, YT: carried out data acquisition and data analysis. All authors read and approved the final manuscript.
